# Do mini-fragment T-plates allow for more proximal rafting than pre-contoured anatomic plates in lateral split-depression tibial plateau fractures?

**DOI:** 10.1007/s00590-025-04435-w

**Published:** 2025-07-26

**Authors:** Elizabeth Lechtholz-Zey, Michael Allen, Ivan Luu, Ian Hasegawa, Joseph Patterson, Joshua Gary

**Affiliations:** 1 Department of Orthopaedic Surgery, University of Southern California, Los Angeles, USA; 2 Department of Orthopaedic Surgery, University of California, San Francisco in Fresno, Fresno, USA

**Keywords:** Tibial plateau, Fracture, Split-depression, Mini-fragment plate, Anterolateral plate, Screw joint distance

## Abstract

**Purpose:**

To compare the proximity of rafting screws to the articular surface in lateral split-depression (LSD) tibial plateau fractures using mini-fragment (MF) and pre-contoured anterolateral (AL) proximal tibia plates. Secondary aims included comparing patient-reported outcome scores and rates of hardware failure and reoperation.

**Methods:**

*Design* Retrospective review. *Setting***:** Multi-center Level I Tertiary Academic Center. *Patient Selection Criteria***:** Adult patients with AO/OTA 41B3.1 LSD tibial plateau fractures treated over 30 months by a single surgeon were identified. Patients were included when their fractures were treated with either a MF T-plate (2.7 mm in thickness) or a pre-contoured anatomic AL plate. *Main Outcome Measures***:** The primary outcome was the distance from the lateral joint line to the most proximal screw (screw-to-joint distance [SJD]) as measured on intraoperative fluoroscopy.

**Results:**

Twenty-four patients were included with patients having received either a 2.7-mm MF T-plate (n = 14) or a standard AL proximal tibia plate (n = 10) and were followed for a median time of 29 weeks. Average SJD was 3.79 mm in the MF group and 8.67 mm in the AL group (p < 0.001). There was no difference in PROMIS scores between the groups. No patients experienced loss of reduction, implant loosening/failure, reoperation, or removal of tibial plateau hardware.

**Conclusions:**

Mini-fragment plates allow for a significantly shorter SJD compared to AL plates, allowing surgeons to provide more proximal rafting of LSD fractures while maintaining low rates of postoperative complications. No increase in fracture subsidence was observed when using mini-fragment fixation alone compared to AL plates.

## Introduction

Tibial plateau fractures can present with various patterns depending on the mechanism of injury and directed force. Lateral split-depression (LSD) tibial plateau fractures are the most common pattern seen [1]. The goals of fracture treatment include restoring the coronal and sagittal alignment with reduction and stabilization of osteochondral fragment(s). Operative management of osteochondral depression involves elevation and rafting of the depressed articular surface [2]. Pre-contoured periarticular buttress plates are frequently used, but individual patient osteology may require these implants to have additional proximal rafting screws below the subchondral bone of depressed fragments outside of the buttress plate. Many surgeons have employed the use of mini-fragment “rim” plates above pre-contoured plates to gain rafting fixation in the sub-chondral bone of osteochondral impaction [4].

Depressed articular fragments are particularly susceptible to subsidence over time due to discontinuity with other fragments and lack of subchondral bone support^4^. Rafting constructs stabilize these fragments, and cancellous bone autograft, allograft chips, or synthetic ceramic cements backfill voids in metaphyseal bone to minimize the risk of recurrent subsidence [5, 6]. Biomechanical studies have demonstrated the superiority of this approach over isolated buttressing techniques in resisting depression from axial loading [7]. Subchondral rafting can be performed using individual lag screws, Kirschner wires, inside–out screws, or a combination of plates and screws [8–11].

Pre-contoured anatomic anterolateral (AL) proximal tibia plates can be prominent under the iliotibial band, resulting in discomfort and irritation that may benefit from implant removal after fracture healing [12, 13]. Pre-contoured anatomic plates in other subcutaneous metaphyseal regions can have similar clinical results. Over the last two decades, orthopedic trauma surgeons began to use lower-profile implants with mini-fragment screws (2.7 mm diameter and below) to manage various fracture types as screws of longer lengths became commercially available [14–16]. The flexibility of the mini-fragment (MF) plates allows for custom contours and cutting the plate to appropriate length to fit the individual patient and fracture. It was hypothesized that 2.7-mm MF T-plates would allow for subchondral rafting in LSD tibial plateau fractures closer to the articular surface than standard pre-contoured anatomic AL proximal tibia plates.

## Patients and methods

Institutional review board approval was obtained to retrospectively identify patients receiving open reduction and internal fixation (ORIF) of closed AO/OTA 41B3.1 unicondylar LSD tibial plateau fractures between October 2021 and December 2023 [17]. The case log from a single surgeon at two large academic institutions was reviewed for current procedural technology (CPT) code 27535. Patients were included if treated with a MF T-plate, defined as 2.7 mm in thickness with 2.7-mm cortical or locking screws, or an anatomic AL plateau plate with associated 3.5-mm screws. All patients with either of the fixation constructs were included in the study. Patients were excluded if they received an alternate implant, sustained an open fracture, or sustained a fracture other than AO/OTA 41B3.1 (Schatzker II) [18]. The Stryker VariAx 2 Mini Fragment system (Stryker, Kalamazoo, MI) was used for MF plating, with a 5-hole (proximal row) T-plate cut to an appropriate length. AL plates were either Synthes 3.5-mm proximal tibia variable-angle lateral compression plate (DePuy Synthes, Raynham, MA) or Stryker AxSOS 3 Ti 4-mm plate (Stryker, Kalamazoo, MI). Patient demographics, injury characteristics, surgical data, and outcomes were obtained through the electronic medical record and picture archiving and communications system (PACS). The primary outcome of interest was the screw-to-joint distance (SJD), measured as the distance from the most proximal rafting screw to the lateral joint line. Due to variations in the standardization of follow-up radiographs obtained in clinic, SJD was measured using final intraoperative fluoroscopy. Measurements were recorded by two observers, an orthopedic trauma fellow and an orthopedic resident. Measurements were taken using Synapse PACS software V7.3.000(FUJIFILM Healthcare Americas Corporation, Lexington, MA). A straight vertical line was drawn from the top of the most proximal screw to the most concave portion of the lateral tibial plateau on AP fluoroscopy using the PACS ruler tool. This measurement was calibrated based on the known screw diameter of 2.7 or 3.5 mm. Radiographs were obtained at 6 weeks post-op and at each follow-up visit to assess for healing, displacement, and/or loss of reduction. Patient-Reported Outcomes Measurement Information System (PROMIS) scores were recorded when available. At each follow-up visit, active and passive knee range of motion and stability were assessed on physical examination.

### Surgical technique

Under general anesthesia, patients are placed supine on a radiolucent operating table. A pneumatic thigh tourniquet is routinely used. A standard anterolateral approach to the proximal tibia proceeds with a curvilinear incision centered over Gerdy's tubercle. The fascia of the iliotibial band is incised, and the anterior compartment musculature is elevated off the proximal tibia distally. A sub-meniscal arthrotomy is performed, and the superior leaf of the capsule is tagged for later repair using #0 Vicryl inside–out vertical mattress sutures. The meniscus is examined for any tears or significant fraying and treated with repair or rarely debridement based upon the complexity and location of the tear [19].

A femoral distractor or manual varus force is used to better evaluate the articular surface prior to osteochondral fragment mobilization and reduction. Depending upon the location of the lateral split and/or chronicity of the injury, a bone tamp through a proximal tibial corticotomy or direct visualization and reduction with external rotation of the split fragment are used to elevate depressed osteochondral fragments. Osteochondral fragments are provisionally stabilized with multiple double-ended Kirschner wires driven from lateral to medial until flush with the lateral edge of the depressed fragment. Autograft or allograft chips are then impacted into the metaphyseal void to serve as structural support for the articular osteochondral fragments.

**Fig. 1:**
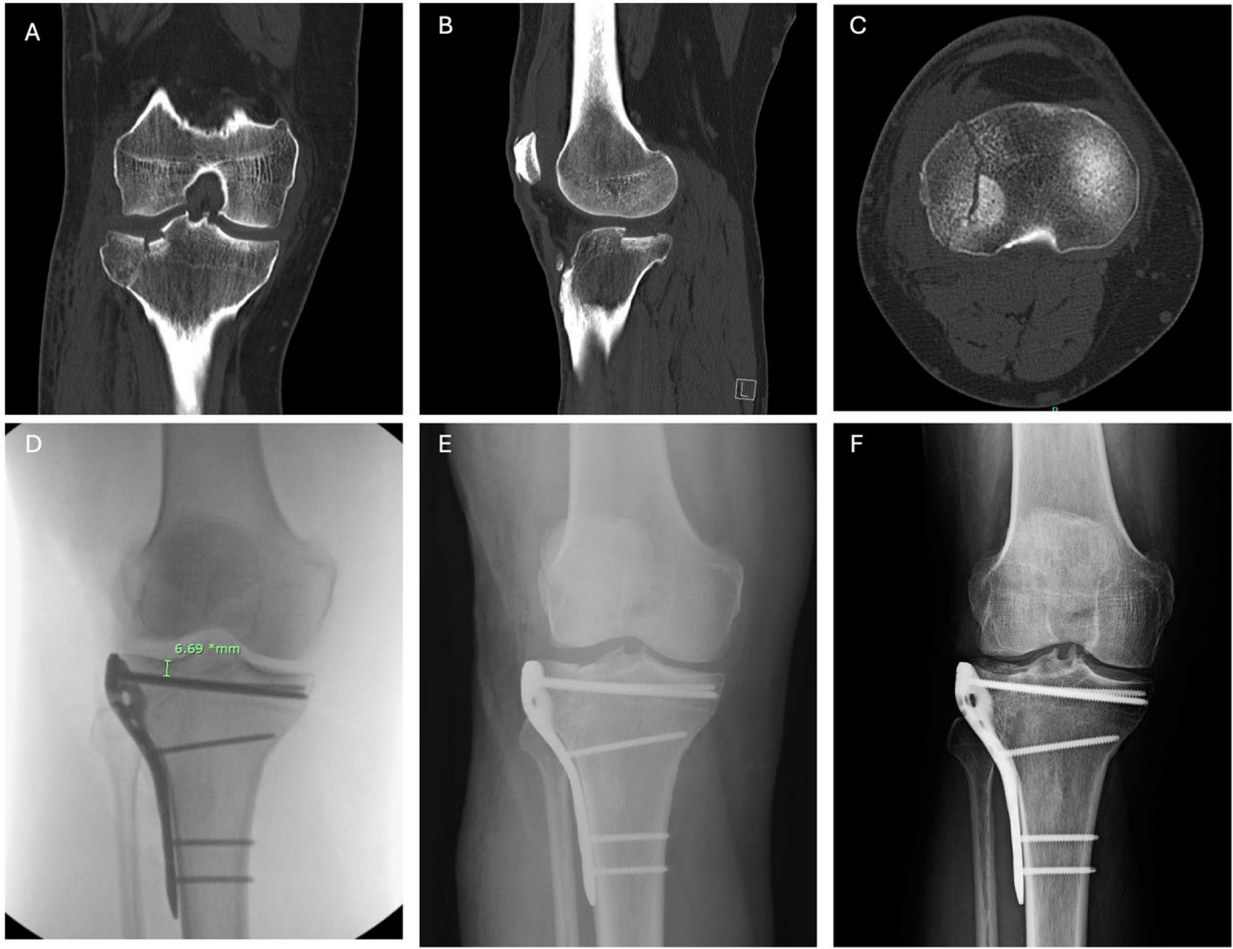
A 60-year-old male sustained this fracture after a ground-level fall and received a standard 3.5-mm anterolateral plate. Preoperative computed tomography demonstrates a right tibial plateau lateral split-depression fracture in the **a** coronal, **b** sagittal, and **c** axial planes. **d** The SJD, as measured on intraoperative fluoroscopy, is 6.69 mm. **e** Immediately postoperatively. **f** Final follow-up at 55 weeks postoperatively shows maintenance of articular congruity and healing

**Fig. 2:**
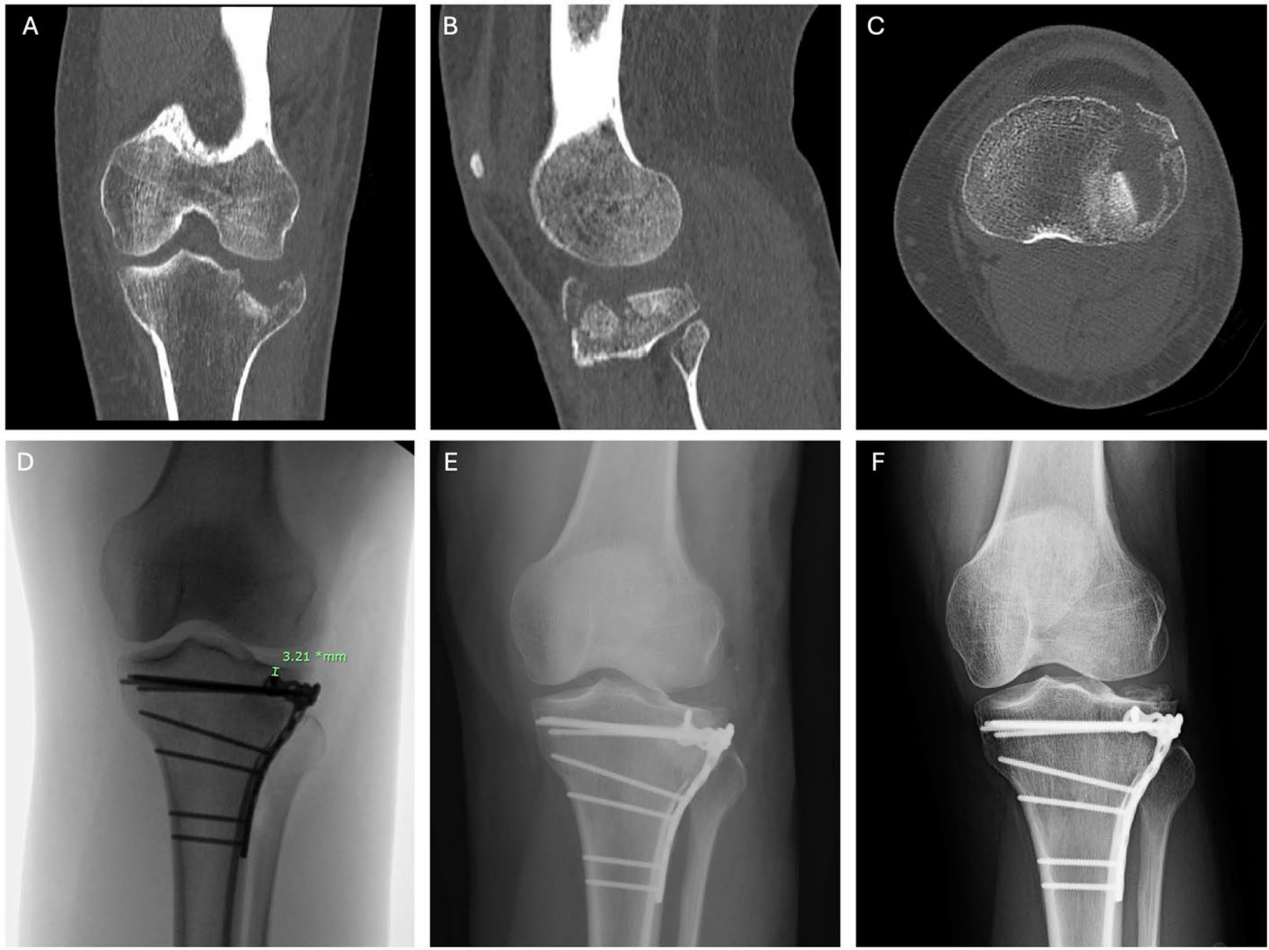
A 42-year-old female fell down stairs and received a 2.7-mm MF T-plate. Preoperative computed tomography demonstrates a left tibial plateau lateral split-depression fracture in the **a** coronal, **b** sagittal, and **c** axial planes. **d** The SJD measured on intraoperative fluoroscopy is 3.21 mm. **e** Immediately postoperatively. **f** Final follow-up at 119 weeks postoperatively shows maintenance of articular healing

The lateral split is then reduced, and the existing Kirschner wires are driven retrograde from medial to lateral through the lateral fragment to raft the joint and hold the lateral plateau reduction. The plate of choice is now selected, contoured if needed, and positioned about the anterolateral surface of the proximal tibia. Precontoured plates are fit in the most proximal position allowed by the manufacturer contour around Gerdy’s tubercle. Pre-contoured anatomic plates are generally chosen for fractures with a lateral split exiting at or distal to the metadiaphyseal region (Fig. 1). A 10-hole T-plate with a 5-hole proximal row is used when an MF plate is selected, and excess length is cut from the plate as needed according to the distal extent of the lateral split. MF T-plates are manually contoured to allow the proximal row to wrap around the anterior plateau, providing anterior-to-posterior and lateral-to-medial vectors for rafting. It is helpful to angle the plate slightly in the sagittal plane to limit screw convergence between the anterior screw and lateral screws (Fig. 2). First, cortical buttress screws are placed distal to the split apex to create a buttress effect for the plate. A large periarticular clamp is then placed with one tine on the medial plateau and the other on the plate to restore condylar width and compress the plate against bone. Cortical or locking screws are placed through the proximal row to raft and maintain the elevation of the depressed fragment. Provisional Kirschner wires are removed and not retained for rafting. All patients received 24 h of antibiotic therapy with cefazolin postoperatively and pharmacologic anticoagulation. Patients were made non-weight bearing to the operative extremity for eight weeks with free range of motion. Radiographs were obtained at 6 and 12 weeks after surgery.

### Statistical analysis

Retrospectively collected data were managed with Microsoft Excel (Microsoft Corporation, Redmond, WA). Statistical analysis was performed using R Statistical Software (v4.3.3; R Core Team 2024). Wilcoxon rank sum test was employed to analyze non-normally distributed continuous variables, and Chi-squared or Fisher's exact tests were used for categorical variables. A two-way random effect model was used to calculate intraclass correlation coefficient (ICC) for SJD measurement. The significance level was set at *p* < 0.05.

## Results

A total of 26 cases were identified. One 41C fracture and one open fracture were excluded leaving 24 patients with 24 lateral tibial plateau fractures. The median age was 41 (IQR 29, 53), 12 (50%) were females, and the median body mass index was 26.7 kg/m^2^ (Table 1). All patients sustained AO/OTA 41B3.1 (Schatzker II) plateau fractures. Patients received either a 2.7-mm MF T-plate (n = 14) or a standard AL proximal tibia plate (n = 10) [Table 2]. Two patients in the AL group received a supplementary proximal 2.4-mm rim plate (one Synthes 4-hole 2.4-mm lateral compression plate [DePuy Synthes, Raynham, MA] and one Stryker 4-hole 2.4-mm plate [Stryker, Kalamazoo, MI]) given the need for more proximal rafting fixation. These patients were kept in the AL group for analysis purposes. Plate and screw type were chosen at the discretion of the attending surgeon based upon fracture pattern and distal extent of the lateral split. The senior author gradually moved toward using MF 2.7-mm T-plates as the plates and appropriate 2.7-mm cortical and locking screw lengths were released onto the market. There were no definitive indications in this retrospective series, but MF 2.7-mm T-plates are generally used when the lateral split exits within 1 cm of the distal portion of Gerdy’s tubercle. Twenty-two patients (92%) received allograft chips or autograft to fill the metaphyseal void and support elevated osteochondral fragments. Six patients sustained additional bony injuries, though all Injury Severity Scores (ISS) were below 15. Twelve patients sustained concomitant ipsilateral meniscal injuries, with nine of these requiring simultaneous intraoperative repair. One patient was initially placed in an external fixator after 4-compartment fasciotomies, with definitive fixation using an AL plate taking place one week later. A second patient presented to the senior surgeon with a missed compartment syndrome and underwent 2-compartment fasciotomies with debridement of nonviable anterior and lateral compartments, ORIF, and primary closure at the index surgery. After surgery, patients were followed for a median of 29 weeks (IQR 19, 75).

**Table 1:** Patient demographics and surgical characteristics

Characteristic	Overall, N = 24^*1*^	Fixation construct		p-value
		AL, N = 10^a^	MF, N = 14^*1*^	
Age	41 (29, 53)	40 (35, 54)	43 (27, 47)	0.5^b^
BMI	26.7 (24.0, 31.6)	28.3 (26.7, 32.9)	25.3 (23.7, 28.6)	0.2^b^
*Sex*				0.10^c^
F	12 (50%)	3 (30%)	9 (64%)	
M	12 (50%)	7 (70%)	5 (36%)	
Tobacco Use	6 (25%)	4 (40%)	2 (14%)	0.2^d^
*ASA*				0.8^d^
1	5 (21%)	2 (20%)	3 (21%)	
2	16 (67%)	6 (60%)	10 (71%)	
3	3 (13%)	2 (20%)	1 (7.1%)	
*Mechanism*				0.8^d^
AVP	4 (17%)	2 (20%)	2 (14%)	
Fall down stairs	2 (8.3%)	1 (10%)	1 (7.1%)	
FFH	3 (13%)	0 (0%)	3 (21%)	
GLF	8 (33%)	4 (40%)	4 (29%)	
Impact	1 (4.2%)	0 (0%)	1 (7.1%)	
MCC	2 (8.3%)	1 (10%)	1 (7.1%)	
MVC	1 (4.2%)	1 (10%)	0 (0%)	
Sporting	3 (13%)	1 (10%)	2 (14%)	
Polytrauma	6 (25%)	4 (40%)	2 (14%)	0.2^d^
Meniscus Injury Repaired	10 (42%)	5 (50%)	5 (36%)	0.7^d^
Fasciotomy	2 (8.3%)	2 (20%)	0 (0%)	0.2^d^
Days Until Surgery	14 (10, 25)	15 (9, 19)	14 (10, 28)	0.7^2^

AVP, Auto vs. Pedestrian; FFH, Fall From Height; GLF, Ground Level Fall; MCC, Motorcycle Collision; MVC, Motor Vehicle Collision

^a^Median (IQR); n (%)

^b^Wilcoxon rank sum test

^c^Pearson's Chi-squared test

^d^Fisher's exact test

**Table 2:** Patient characteristics for tibial plateau fractures treated with anterolateral and mini-fragment plates

Age	Sex	BMI	Tobacco	Comorbidities	ASA	Mechanism	Polytrauma	Days to Surgery	Fixation	SJD (mm)	ROM (°)	Follow-up (weeks)	Complications
59	F	27.4	No	Asthma, Bronchitis	2	FFH	No	13	MF	2.515	140	30.1	None
26	F	21.0	No	None	1	Impact	No	33	MF	5.330	140	20.9	None
55	F	19.1	No	None	2	GLF	No	18	AL	9.205	140	28.1	None
45	F	32.2	No	Major Depressive Disorder (MDD)	2	GLF	No	10	MF	5.655	140	85.3	None
29	M	29.0	Yes	Prior Tibial Shaft Fracture	2	GLF	No	40	MF	3.790	140	64.1	None
60	M	33.4	No	Prediabetes, Dyslipidemia, Polycythemia	2	GLF	No	6	AL	7.720	140	81.3	None
27	M	26.6	No	None	2	MVC	Yes	8	AL	7.960	140	104.1	None
25	F	26.2	Yes	None	1	Sporting	No	24	MF	2.880	140	52.3	Superficial infection
37	M	31.4	No	None	1	GLF	No	34	AL	12.155	140	122.3	None
42	F	21.3	No	Anxiety, Hypothyroidism, MDD	2	Fall down stairs	No	8	MF	3.765	140	119.1	None
70	F	24.2	No	Asthma, MDD, Hyperlipidemia	2	FFH	No	17	MF	6.730	140	21.1	Preoperative compartment syndrome with common peroneal palsy
23	M	23.1	No	None	2	MCC	No	42	MF	4.350	140	12.1	None
21	M	25.1	No	None	2	AVP	Yes	10	MF	2.575	140	47.3	None
52	M	33.6	Yes	Obesity	2	MCC	Yes	49	AL	7.765	130	20.0	Preoperative deep peroneal palsy
32	M	43.9	Yes	Obesity, Opioid Abuse	2	GLF	No	12	AL	8.505	110	115.1	Rigidity/stiffness
64	F	26.8	No	Breast Cancer, Osteopenia, Prediabetes	3	Fall down stairs	No	19	AL	3.595	80	23.9	Rigidity/stiffness
40	M	28.1	Yes	None	3	Sporting	No	6	AL	12.390	90	2.1	None
40	F	28.5	No	Overweight	2	AVP	Yes	19	AL	10.785	135	70.0	None
57	F	23.6	No	None	2	GLF	No	10	MF	6.655	135	14.9	None
28	M	25.1	No	Crohn's Disease, MDD, Anxiety	1	Sporting	No	11	MF	3.360	90	2.0	Preoperative deep peroneal palsy
34	M	22.6	Yes	None	1	AVP	Yes	10	AL	8.830	85	26.1	None
45	M	33.4	No	Obesity	2	FFH	No	8	MF	4.285	135	11.0	None
44	F	36.7	No	Hypertension, Obesity	2	GLF	No	29	MF	3.010	90	10.0	None
48	F	25.4	No	Hyperlipidemia, Pulmonary Embolism	3	AVP	Yes	15	MF	NA	140	73.3	Rigidity/stiffness

AL, Anterolateral Plate; AVP, Auto vs. Pedestrian; FFH, Fall From Height; GLF, Ground Level Fall; MCC, Motorcycle Collision; MF, Mini-fragment Plate; MVC, Motor Vehicle Collision

The median distance from the most proximal rafting screw to the lateral joint line (screw-to-joint distance [SJD]) on intraoperative fluoroscopy was 3.79 mm (IQR: 3.01–5.33) in the MF group and 8.67 mm (IQR: 7.81–10.39) in the AL group (*p* < 0.001) [Table 3]. For the two AL patients who received supplementary rim plates, the SJD was measured from the AL plate screws. Two independent raters performed the SJD measurements, which are reported as the mean value. The ICC was 0.9, with a *p*-value of < 0.001, indicating excellent agreement.

**Table 3:** Post-operative outcomes for all included patients

Characteristic	Overall, N = 24^*1*^	Fixation Construct		p-value
		Anterolateral, N = 10^a^	Mini-fragment, N = 14^*1*^	
Length of Follow-up (weeks)	29 (19, 75)	49 (24, 98)	26 (13, 61)	0.3^b^
Screw Joint Distance (mm)	5.66 (3.68, 8.23)	8.67 (7.81, 10.39)	3.79 (3.01, 5.33)	< 0.001^b^
Estimated Blood Loss (mL)	48 (25, 53)	50 (30, 75)	43 (25, 50)	0.2^b^
Operative length (minutes)	129 (118, 157)	148 (127, 178)	123 (115, 135)	0.069^b^
*Bone Graft*				0.7^c^
Allograft	21 (88%)	8 (80%)	13 (93%)	
Autograft	1 (4.2%)	0 (0%)	1 (7.1%)	
None	2 (8.3%)	2 (20%)	0 (0%)	
Final Range of Motion (°)	140 (132.5, 140)	135 (110, 140)	140 (137.5, 140)	0.17^b^
Rigidity/Stiffness	3 (13%)	1 (10%)	2 (14%)	> 0.9^c^
Infection				0.4^c^
None	23 (96%)	9 (90%)	14 (100%)	
Superficial	1 (4.2%)	1 (10%)	0 (0%)	
PROMIS Score Available	9 (39%)	2 (22%)	7 (50%)	0.21^c^
Time of PROMIS (weeks postop)	25 (19.9, 46.4)	78.5 (41, 116)	22.4 (18.3, 42.9)	0.221^b^
Percent of Normal	64 (51, 90)	72.5 (60, 85)	64 (50, 95)	0.88^b^
Global Physical Health	44.9 (41.1, 50.8)	48.2 (42.3, 54.1)	44.9 (39.8, 47.4)	0.77^b^
Global Mental Health	43.5 (36.3, 49.6)	41.1 (36.3, 45.8)	43.5 (36.3, 50.8)	0.77^b^
Pain Interference	56.4 (48.7, 62.2)	55 (50.3, 59.6)	56.4 (48.7, 62.2)	0.89^b^
Physical Function	44.6 (41.2, 51.7)	48.8 (47.4, 50.2)	41.2 (37.6, 51.7)	0.46^b^

No patient lost reduction or fixation postoperatively

^a^Median (IQR); n (%)

^b^Wilcoxon rank sum test

^c^Fisher's exact test

For the functional outcomes, the two patients who received supplementary rim plates in addition to the AL plates were excluded from the final analyses, as well as the two patients who did not receive bone grafting in order to mitigate heterogeneity. The median passive range of motion was 0-140º in the MF group and 0-135º in the AL group (p = 0.17). One patient with an AL plate had knee stiffness not requiring intervention, while two patients who received the MF plate and one who received the AL plate had clinically significant knee stiffness/rigidity that required treatment with manipulation under anesthesia given lack of improvement with physical therapy within 8 weeks of index procedure. One patient who received an AL plate had a superficial wound infection that resolved with oral antibiotic therapy. There were no cases of reoperation or removal of tibial plateau hardware. PROMIS scores were available for ten patients (3 AL and 7 MF), though one patient in the AL group was excluded given that they received an additional rim plate. Median pain interference was 55 (IQR 50.3, 59.6) for AL and 56.4 (IQR 48.7, 62.2) for MF (*p* = 0.89). Median physical function was 48.8 (IQR 47.4, 50.2) for AL and 41.2 (IQR 37.6, 51.7) for MF (*p* = 0.46). No patients experienced loss of reduction, implant loosening, or implant failure on any post-operative radiographs.

## Discussion

This study reports that MF 2.7-mm T-plates allowed for subchondral rafting in LSD (Schatzker II) tibial plateau fractures closer to the articular surface than pre-contoured anatomic lateral proximal tibia plates. MF T-plates with 5 holes on the proximal row also allowed for multiplanar rafting screws with lateral to medial and anterior-to-posterior trajectories. Newer mini-fragment implants from multiple manufacturers now have 2.7-mm screws up to 80 mm that were not previously available (Smith & Nephew EVOS [Smith & Nephew, Memphis, TN], Stryker VariAx 2 [Stryker, Kalamazoo, MI], Anthem [Globus, Audubon, PA]). At the final follow-up, all patients in both groups demonstrated maintainance of reduction without articular collapse.

Proximal rim plates above pre-contoured anatomic plates have been another successful method to address this issue with excellent results and were employed for two patients in this series treated with pre-contoured anatomic plates [3, 20, 21]. Using a MF 2.7-mm T-plate with a long proximal row combines the benefits of proximal rim rafting with the ability to buttress in one low-profile implant. There were no postoperative complications that required intervention and only one instance of superficial wound infection in a pre-contoured anatomic plate that resolved with oral antibiotic therapy. There was no recurrent displacement or loss of reduction in either group, and PROMIS physical function and pain interference scores were no different, when available.

Over the last several decades, orthopedic surgeons have trended toward using lower-profile plates to fix several different fracture types. Wadwa et al. found that applying a 2.7-mm mini-fragment plate to isolated olecranon fractures resulted in fewer symptomatic hardware instances than precontoured olecranon-specific plates [16]. Prasarn et al. demonstrated that dual mini-fragment plating over the anterior and superior portions of midshaft clavicular fractures resulted in successful fracture union in all patients while avoiding secondary surgeries [15, 22]. In the lower extremity, mini-fragment (≤ 2.8 mm) plates were compared with 3.5-mm small-fragment plates in a cohort of 120 patients with distal fibular fractures. Patients in the mini-fragment group experienced increased rates of anatomic reduction, zero implant complications, and fewer instances of plate removal [14]. Cho et al. described the successful application of a 2.7-mm variable-angle locking compression plate to the rim of posterolateral tibial plateau fractures, along with an AL buttressing plate, with the restoration of the full range of motion in most patients and no loss of reduction during the follow-up period [3]. More recently, Chen et al. used individual small- and mini-fragment plates applied in either a rim or buttressing fashion to fix partial articular tibial plateau fractures in a small case series of 19 patients and reported a 100% union rate with no loss of reduction or need for reoperation due to hardware irritation [21]. Lower-profile plates are thinner and thus more malleable, making them amenable to intraoperative shaping around the most proximal portion of the tibia [3]. Attempts to recontour pre-contoured anatomic locking plates can unpredictably change the trajectory of the screw holes or damage the locking mechanism, which may limit the ability of these constructs to capture depressed fragments due to limited locking screw trajectories [4, 23, 24].

Subchondral rafting screws are an essential aspect of maintaining articular reduction with depressed fragments [25]. The SJD can impact reduced articular surface subsidence in LSDs. Ye et al. assessed 49 Schatzker II-VI fractures with depressed fragments and demonstrated that a smaller SJD was significantly associated with a lower risk of articular subsidence [4]. The present cohort demonstrated that the MF construct allowed for a smaller SJD than the AL group (*p* < 0.001).

This study has limitations, including its retrospective nature, small sample size, and inconsistent follow-up lengths. Plate selection was also based on attending surgeon preference and was not randomized. Our functional outcome data are limited as our public hospital does not have the infrastructure or personnel to routinely collect longer-term follow-up data and thus is likely underpowered to detect meaningful clinical differences. The strengths of this study include single surgeon consistency in technique and application. This study is one of the first to focus specifically on comparing the distance from the most proximal rafting screw to the lateral joint line between mini-fragment and standard anatomic plates.

## Conclusion

Mini-fragment plates can significantly decrease the SJD when compared with standard AL plates, allowing surgeons to provide more proximal rafting of LSD fractures. While our series demonstrated a low rate of postoperative complications, larger series with longer follow-up are needed to determine functional outcomes and longer-term complications. No increase in fracture subsidence was observed when using mini-fragment fixation compared to AL pre-contoured plates.

## Data Availability

No datasets were generated or analysed during the current study.
